# Technology affinity, digital tool use, and implications for health professions education: a cross-sectional study

**DOI:** 10.1186/s12909-026-09465-4

**Published:** 2026-05-20

**Authors:** Alina Susanne Unkart, Eva Decker, Nils Lahmann, Sandra Strube-Lahmann

**Affiliations:** 1 Klinik für Geriatrie und Altersmedizin, Forschungsgruppe Geriatrie, Charité - University Medicine Berlin, Berlin, Germany; 2 Medical School Berlin, Berlin, Germany; 3 Stabsstelle Pflegewissenschaft, Universitätsmedizin Göttingen, Göttingen, Germany

**Keywords:** Digital health education, Technology affinity, Digital transformation, Digital readiness, Nursing

## Abstract

**Background:**

Digital transformation in healthcare requires professionals who are prepared to integrate technological innovations into clinical practice. Beyond structural implementation, successful adoption depends on individual technology affinity and everyday digital engagement. However, evidence on how routine digital tool use relates to technology attitudes among healthcare professionals remains limited. This study examined the association between daily digital tool usage and technology affinity in German healthcare professionals and explored implications for education and training.

**Methods:**

An exploratory quantitative cross-sectional survey was conducted in accordance with the STROBE reporting guidelines. An anonymous online questionnaire was administered between June 2023 and April 2024 among healthcare professionals in Germany. Technology affinity was measured using the validated TAEG scale, assessing enthusiasm for technology, perceived competence, perceived benefits, and perceived drawbacks. Statistical analyses comprised descriptive statistics and group comparisons. To examine differences in technology affinity across levels of daily digital tool usage while controlling for potential confounders, a multivariate analysis of covariance (MANCOVA) was conducted with age, gender, education, and profession as covariates. In addition, multiple regression analyses were performed to assess independent associations between digital tool usage and technology affinity dimensions. All analyses were conducted using IBM SPSS Statistics version 29.

**Results:**

Data from 1,211 healthcare professionals were analyzed. Higher levels of daily digital tool usage were significantly associated with stronger technology affinity across all dimensions, independent of sociodemographic and professional factors. Younger and male participants demonstrated higher affinity scores. Differences between nurses and other healthcare professionals were small; however, given the aggregated nature of the occupational variable, these findings should be interpreted with caution and do not permit conclusions regarding differences between specific professional groups.

**Conclusions:**

Everyday digital engagement appears to be closely associated with positive technology attitudes among healthcare professionals, although causal relationships cannot be inferred. Educational and professional development programs should therefore integrate structured opportunities for practical digital tool use and address age- and gender-related differences in digital confidence. Strengthening digital competence through experiential learning may enhance readiness for digital innovation in healthcare settings.

## Background

Digital transformation in healthcare is increasingly seen as a structural necessity rather than a technological option. Germany is driving digital transformation in healthcare through a national strategy [1] supported by key legislation, including the Digital Act (DigiG), which introduces an opt-out electronic patient record for all insured individuals, and the Health Data Use Act (GDNG), which facilitates the secondary use of health data for research and innovation.

However, despite considerable investment, adoption rates of digital technologies among frontline healthcare workers remain inconsistent [2–4]. Limited adoption may hinder the effective implementation of digital health innovations, reduce efficiency in care processes, and limit the potential benefits of data-driven healthcare. In addition, insufficient digital readiness may exacerbate disparities in access to digital tools and compromise the sustainability of ongoing transformation efforts [5]. Several reports suggest that, beyond technical infrastructure and training, the attitudes and digital mindset of healthcare professionals, conceptualized in this study as technology affinity, play a crucial role in successful implementation [6–8]. Prior experience with and familiarity across different types of technologies, ranging from well-established systems to more complex or emerging applications, may further shape individuals’ attitudes and readiness to engage with digital innovations [9]. Despite growing conceptual interest in digital attitudes, empirical evidence linking everyday digital usage with multidimensional technology affinity among healthcare professionals, particularly within the German healthcare system, remains limited.

Existing empirical studies in Germany show significant variation in digital readiness across professional groups, regions, and institutional types [10]. Nurses in particular have been shown to experience higher barriers to digital engagement, often due to limited autonomy, insufficient IT support, and lack of structured digital training pathways [3, 4, 11]. At the same time, many nurses report high willingness to adopt digital tools when adequately supported and involved in decision-making [8, 12, 13].

The aim of this study was to examine the association between daily digital tool use and technology affinity among healthcare professionals in Germany. Using the validated multidimensional TAEG instrument [14], the study further explores differences across occupational categories and usage patterns and discusses implications for health professions education and digital workforce development. Technology affinity, as examined in this study, refers to an individual’s general interest in, confidence with, and openness toward digital technologies. The concept is closely related to, but distinct from, classical technology acceptance frameworks such as the Technology Acceptance Model (TAM) [15, 16] and the Unified Theory of Acceptance and Use of Technology (UTAUT) [17, 18]. While those models focus primarily on behavioral intention and perceived usefulness, affinity encompasses underlying attitudes and emotional readiness, regardless of specific systems or contexts [14, 19]. In this regard, it serves as a latent marker for digital maturity at the individual level [20, 21].

Digital tool usage in this study represents a broad proxy for everyday digital exposure. While this limits differentiation between types and complexity of use, it prioritizes ecological validity by capturing routine digital engagement in real-world contexts.

## Methods

### Design

This study employed a quantitative, exploratory cross-sectional survey design, following the STROBE reporting guidelines for observational studies [22]. A cross-sectional design was chosen as an appropriate approach to explore associations between digital tool use and technology affinity in a large and heterogeneous sample, as it enables the identification of patterns and relationships in an underexplored area without requiring longitudinal data.

### Study setting and sampling

Participants were recruited between June 2023 and April 2024. The target population consisted of healthcare professionals employed in Germany across various sectors, including hospitals, outpatient services, long-term care, and rehabilitation. Recruitment was conducted through professional associations, hospital mailing lists, and targeted social media posts. To reduce selection bias, multiple networks were used to reach a diverse group of healthcare professionals. A total of 1,211 responses were included in the analysis after data cleaning for incomplete and implausible responses (defined as > 95% missing values or implausible response patterns (e.g., uniform responses across all items)). Participants with partially incomplete questionnaires were retained if all items required to compute a given TAEG subscale were fully completed. Subscale scores were thus based on complete-case item responses within each subscale, while cases with missing items on a subscale were excluded from analyses involving that subscale. As a result, the number of observations varied slightly across subscales. This approach maximized data use while maintaining internal consistency and reliability of the subscale scores. Since the survey was distributed via open channels without a defined sampling frame, no exact response rate could be determined. The large sample size was considered sufficient to provide precise estimates and enable meaningful subgroup analyses. Due to the open, non-probabilistic sampling, the results are primarily hypothesis-generating.

### Instrument validity and reliability

Technology affinity was assessed using the TAEG questionnaire (Technikaffinität für elektronische Geräte), a validated German-language scale developed by Karrer et al. [19] to ensure measurement validity and reliability. The instrument comprises 19 items across four subscales:

1. *Enthusiasm for technology*,
2. *Perceived competence*,
3. *Perceived benefits*, and
4. *Perceived drawbacks*.

All items were rated on a 5-point Likert scale ranging from 1 (“strongly disagree”) to 5 (“strongly agree”). Cronbach’s alpha values were reported to range from 0.73 to 0.86, confirming satisfactory internal consistency. The “drawbacks” subscale was reverse-coded so that higher values consistently represented greater affinity.

### Data collection

Data were collected anonymously via an online questionnaire using the German online platform *SoSci Survey*, which is widely used in academic and applied research [23]. No incentives were provided, which may have reduced response bias and social desirability effects. Participants provided informed consent before accessing the survey.

### Data analysis

All analyses were performed using IBM SPSS Statistics (Version 29). Participants reported their daily usage time for different categories of digital tools, including smartphone applications, computer programs (e.g., word processing, documentation), and video games. For analysis, these values were aggregated into a single measure of total daily digital tool usage. This composite measure was subsequently dichotomized (≤ 2 h vs. >2 h per day) to facilitate multivariate analysis and ensure adequate group sizes.

For regression analyses, age was also dichotomized (> 40 years vs. ≤40 years) to reflect potential generational differences in digital socialization patterns. Gender (male vs. female), educational attainment (higher education degree vs. other), participation in continuing education within the past 12 months (yes/no, reflecting variation in recent engagement with professional development activities), and professional role (nursing profession vs. other health professions) were included as binary predictors. Professional role was operationalized as a binary variable (nursing profession vs. other health professions) to ensure sufficient group sizes for multivariate analysis. Nurses were used as the reference category due to their relatively large representation in the sample. This approach represents an analytical simplification and does not allow for differentiation between specific health professions or occupation-specific contexts. While dichotomization reduces variability, it is a commonly applied strategy in exploratory analyses with heterogeneous samples to ensure model stability and interpretability of multivariate results. All categorical predictors were dummy coded (1 = characteristic present), and all independent variables were entered simultaneously into the regression models. Gender was originally assessed with multiple response options (female, male, diverse). Due to the very small number of participants selecting “diverse”, gender was dichotomized (male vs. female) for multivariate analyses. Cases indicating “diverse” were excluded from regression and MANCOVA models to ensure statistical stability. Descriptive statistics for all gender categories are reported in Table 1.

Descriptive statistics were used to characterize the sample and summarize affinity scores. For group comparisons, analyses were conducted between nurses and other health professionals, as well as between low and high digital usage groups. Chi-square tests were used for categorical variables, and t-tests or Mann–Whitney U tests for continuous or ordinal variables depending on distribution.

To assess predictors of technology affinity, four multiple linear regression models (one for each TAEG subscale) were conducted. Standardized regression coefficients (β) and exact *p*-values are reported. In addition, a multivariate analysis of covariance (MANCOVA) tested the joint influence of digital usage intensity on all four subscales simultaneously, controlling for age, gender, education, continuing education participation, and professional role.

All statistical tests were two-sided with a significance level of *α* = 0.05. Exact *p*-values are reported, truncated to three decimal places.

Artificial intelligence–based language tools (ChatGPT, OpenAI) were used to support English language refinement. All content was critically reviewed and approved by the authors, who take full responsibility for the manuscript.

## Results

### Characteristics of the sample

The final sample consisted of 1,211 healthcare professionals, of whom 49% were nurses, with the remainder comprising therapists, medical assistants, and other health care professions (summarized in Table 1). The average age was 38.9 years (*SD* = 11.8), ranging from 18 to over 60 years, and 70.7% of respondents identified as female. A summary of participant characteristics is presented in Table 1. 

**Table 1:** Descriptive characteristics of the sample (*n* = 1,211)

Variable	Value
Age (mean, SD)	38.9 (11.8)
Gender: Female (%)	70.7%
Gender: Male (%)	28.2%
Gender: Diverse (%)	1.1%
Profession: Nurses (%)	48.6%
Profession: Other health professions (%)	51.4%

### Group comparisons

**Fig. 1:**
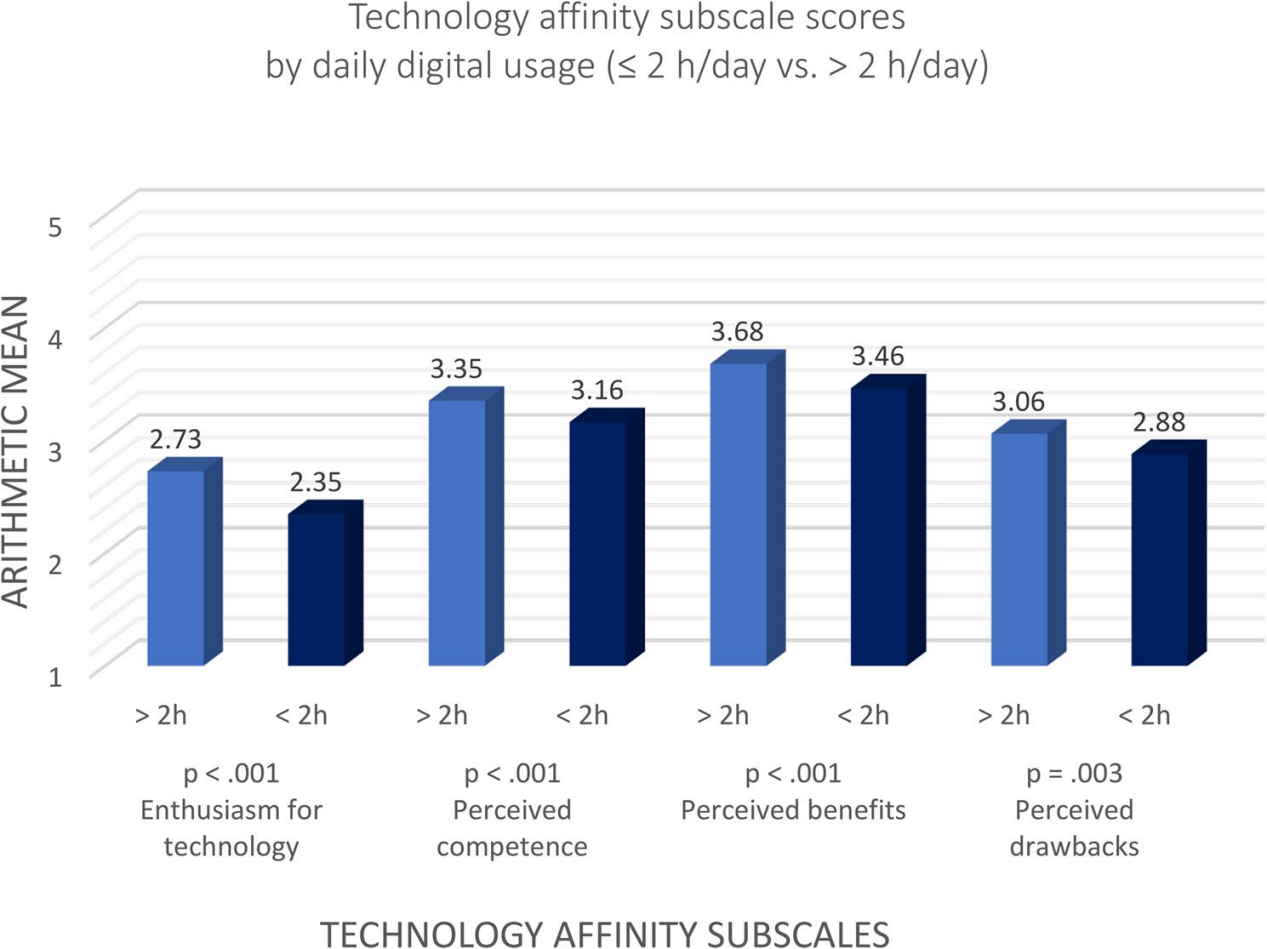
Technology affinity subscale scores by daily digital usage (≤ 2 h/day vs. > 2 h/day)

Figure 1 presents the mean scores of the four TAEG subscales, stratified by daily digital technology use (< 2 h vs. > 2 h per day). Participants with higher daily usage reported consistently higher scores across all subscales. Group sizes varied slightly across subscales, ranging from *n* = 489 to *n* = 550 for the group with more than two hours of daily use, and from *n* = 279 to *n* = 308 for the group with lower usage.

The most pronounced differences emerged in the domains of Enthusiasm for technology (*M* = 2.73 vs. 2.35; *t*(809) = − 4.67, *p* < .001, *d* = − 0.35), and Perceived competence (*M* = 3.35 vs. 3.16; *t*(766) = − 3.85, *p* < .001, *d* = − 0.29), followed by Perceived benefits (*M* = 3.68 vs. 3.46; *t*(856) = − 3.97, *p* < .001, *d* = − 0.28). Interestingly, frequent users also reported slightly higher scores on Perceived drawbacks (*M* = 3.06 vs. 2.88; *t*(827) = − 2.94, *p* = .003, *d* = 0.21), suggesting a more nuanced or differentiated experience with technology in this group.

**Fig. 2:**
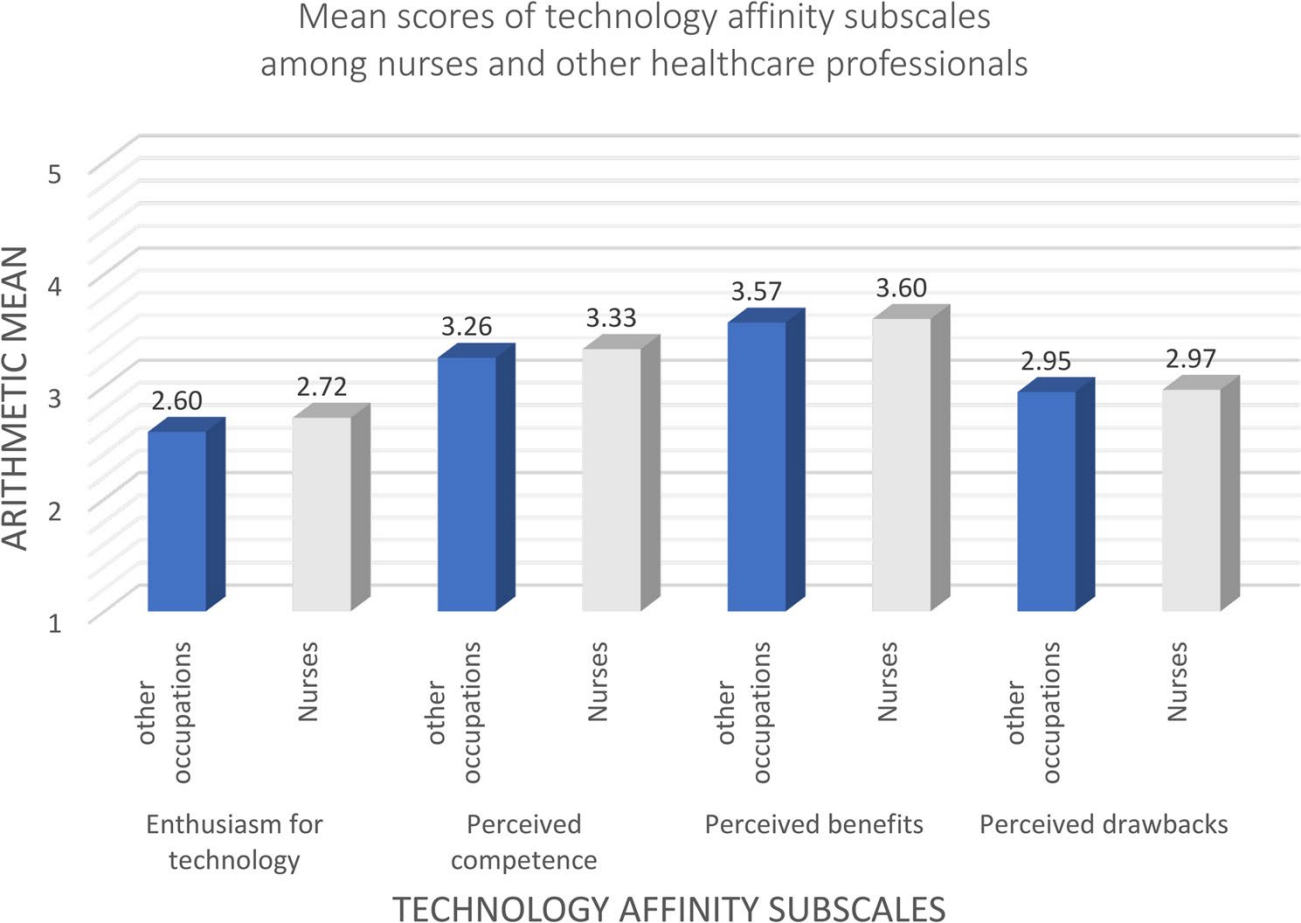
Mean scores of technology affinity subscales among nurses (*n* = 485–543) and other healthcare professionals (*n* = 510–572)

Figure 2 compares affinity scores between nurses and other healthcare professionals. While overall differences were small, nurses scored slightly higher on all four Scales. Inferential statistical testing using t-tests revealed no significant group differences for any of the subscales (*p* > .05).

Due to the binary operationalization of professional role, these comparisons reflect differences between nurses and a heterogeneous group of other health professions and do not allow for more fine-grained occupation-specific interpretations.

### Regression analysis

Digital tool usage was the only predictor that demonstrated consistent significant associations across all technology affinity subscales. In contrast, other variables (e.g., age and gender) were significantly associated with specific subscales only as shown in Table 2. All four regression models predicting the TAEG subscales were significant (all *p* < .05).

**Table 2:** Multivariable linear regression analyses of sociodemographic and professional predictors of technology affinity dimensions

	Enthusiasm		Perceived competence		Perceived benefits		Perceived drawbacks	
	Std. β	*p*	Std. β	*p*	Std. β	*p*	Std. β	*p*
Age > 40 years	-0.12	< 0.001	-0.19	< 0.001	-0.13	< 0.001	0.04	0.276
Male	0.23	< 0.001	0.16	< 0.001	0.07	< 0.001	0.02	0.521
Higher education degree	-0.01	0.807	-0.02	0.542	0.02	0.542	-0.05	0.193
Continuing education	0.06	0.083	-0.01	0.689	0.06	0.689	0.02	0.561
> 2 h digital tool usage	0.14	< 0.001	0.11	0.002	0.11	0.002	-0.09	0.012
Nursing profession	0.00	0.913	0.04	0.259	0.01	0.259	-0.04	0.258

Std. β = standardized regression coefficient. All predictors were entered simultaneously. Binary variables were dummy coded (1 = characteristic present). Reference categories: ≤ 40 years, female, no higher education degree, no continuing education, ≤ 2 h digital tool usage, other health professions

Given that the TAEG subscales represent interrelated dimensions of technology affinity, a multivariate analysis of covariance (MANCOVA) was conducted to account for their intercorrelation, to examine the combined attitudinal profile across subscales, and to reduce the risk of Type I error inflation associated with multiple univariate analyses. Gender, educational attainment, continuing education, and usage duration (≤ 2 h vs. >2 h) were included as fixed factors, while age was entered as a covariate. Assumptions for MANCOVA were evaluated. Homogeneity of covariance matrices was supported (Box’s *M* = 156.752, *F*(130, 8044.549) = 1.064, *p* = .295), and Levene’s tests indicated homogeneity of error variances for all dependent variables (all *p* > .05). Pillai’s trace was used for multivariate inference due to its robustness.

Based on Pillai’s trace, significant multivariate effects were observed for gender, *V* = 0.082, *F*(4, 630) = 14.15, *p* < .001, age, *V* = 0.046, *F*(4, 630) = 7.63, *p* < .001, usage duration, *V* = 0.032, *F*(4, 630) = 5.15, *p* < .001, and continuing education, *V* = 0.016, *F*(4, 630) = 2.50, *p* = .041. Educational attainment showed no multivariate effect, *V* = 0.006, *F*(4, 630) = 0.88, *p* = .475. Multivariate effects (Pillai’s V) were small to moderate, with gender showing the largest multivariate effect.

Follow-up univariate ANCOVAs indicated that gender was associated with higher enthusiasm, competence, and positive attitudes (all *p* ≤ .012), but not negative attitudes. Age > 40 years was related to lower enthusiasm, competence, and positive attitudes (all *p* ≤ .014). Greater usage duration (> 2 h) was associated with higher enthusiasm, competence, and positive attitudes as well as lower negative attitudes (all *p* ≤ .040). Continuing education showed a small effect limited to enthusiasm (*p* = .014), this discrepancy compared to the multivariate regression models may reflect differences in model specification and shared variance with other predictors.

## Discussion

### Summary of findings

This study examined the relationship between daily digital tool usage and technology affinity among healthcare professionals in Germany. The inclusion of entertainment-oriented digital activities, such as video gaming, reflects a broad conceptualization of digital exposure. While such activities may not directly relate to professional tasks, they may still contribute to general digital familiarity and confidence. Higher usage duration was associated with a more favorable overall attitudinal profile, reflected in higher enthusiasm, competence, and perceived benefits, as well as lower perceived drawbacks. These associations remained significant after controlling for age, gender, educational attainment, and continuing education. While nursing professionals showed slightly higher scores across the four dimensions, these differences were small in magnitude. Demographic patterns were also observed: younger participants reported more positive attitudes and higher perceived competence, whereas gender differences primarily affected positive attitudinal dimensions rather than negative perceptions.

### Interpretation in context

Our findings corroborate previous research suggesting that repeated, meaningful interaction with digital tools is associated with more positive affective and cognitive orientations toward technology [10, 24, 25]. In contrast to classical acceptance models such as TAM or UTAUT, which emphasize intention to use specific technologies [15, 16], the concept of technology affinity encompasses a broader, trait-like readiness to engage with digital innovations [19]. Crucially, this readiness appears to evolve through habitual use and positive experiences, particularly when users feel competent and perceive tangible benefits [21, 26].

In line with recent large-scale studies on healthcare professionals (e.g [21, 24]), , our results indicate that variations in digital affinity were not strongly differentiated by professional role within the analytical framework of this study and more closely linked to demographic characteristics. Specifically, increasing age and female gender were associated with lower scores across several dimensions of technology affinity. This pattern aligns with previous research emphasizing the moderating role of sociodemographic factors in digital engagement, particularly within nursing populations [3, 27]. Importantly, these differences should not be interpreted as inherent deficits, but rather as the result of unequal exposure to digital technologies, differential opportunity structures, and disparities in access to training and support. Within the analytical framework of this study, no pronounced differences were observed between nurses and the aggregated group of other health professionals. However, given the binary operationalization of professional role, these findings should be interpreted with caution and do not allow conclusions regarding specific professions. The absence of observed differences should therefore not be interpreted as evidence of equivalence, but rather as a consequence of the aggregated analytical approach.

Our findings suggest that digital competence development should move beyond one-time training sessions toward longitudinal, experience-based learning formats integrated into undergraduate and continuing health professions education. Technology affinity profiling may help educators identify learners who require differentiated instructional approaches. From a policy perspective, digital transformation initiatives in healthcare should therefore adopt inclusive strategies that explicitly address age- and gender-related disparities in digital experience and confidence, in order to promote equitable participation in technological change [28, 29].

### Strengths and limitations

This study benefits from a large and diverse sample, the use of a validated instrument (TAEG), and multivariate analysis techniques. However, several limitations should be noted. First, the study employed a cross-sectional self-report design, which limits causal interpretations and may be subject to socially desirable responding [14]. Second, the TAEG instrument used was the original version [19], which captures more established technologies and may not fully reflect attitudes toward newer digital tools. Third, the number of independent variables included in the models was limited, hence it is possible that other factors such as organizational conditions or cultural attitudes—may play a significant role [2]. Longitudinal and mixed-methods studies are especially needed to unpack how contextual enablers—such as digital leadership, organizational culture, and time budgets—moderate the relationship between digital affinity and actual adoption behavior [2, 4, 28]. While the effect sizes were rather small, the high level of statistical significance—based on a large sample (*N* = 1211)—indicates a robust relationship. Even modest effects can carry practical relevance in large-scale or socially significant contexts, particularly when observed consistently across multiple domains [30, 31]. Fourth, the dichotomization of key variables, including digital tool usage and professional role, may have led to a loss of information and reduced the ability to detect more nuanced associations. In particular, the binary operationalization of professional role (nursing vs. other health professions) grouped diverse healthcare occupations into a single comparison category and therefore does not capture heterogeneity in roles, responsibilities, or digital exposure. As such, the study cannot assess whether technology affinity differs systematically between specific professional groups. Although digital tool usage was initially assessed across different categories, the aggregation into a single composite measure does not allow differentiation between types, purposes, or complexity of technology use. For example, the measure does not distinguish between passive, entertainment-oriented use and active, task-oriented or professional applications. This limits the interpretability of the observed associations. The aggregation approach reflects a focus on overall digital exposure as a general behavioral indicator, rather than on specific technology domains. This aligns with the conceptualization of technology affinity as a broad attitudinal construct rather than a domain-specific competence. Finally, the sample was based on an online survey, which could introduce self-selection bias, particularly among digitally confident individuals. Taken together, these limitations primarily affect the granularity—but not the overall direction—of the observed associations.

## Conclusion

This study demonstrates that technology affinity is associated with overall digital tool usage and selected sociodemographic characteristics among healthcare professionals in Germany. Higher levels of digital exposure were consistently linked to more positive attitudes toward technology; however, given the aggregated and dichotomized nature of key variables, these findings should be interpreted as general associations rather than evidence of specific competencies or occupation-related differences.

The results suggest that general digital familiarity may represent a foundational component of readiness for digital transformation, although it does not substitute for context-specific skills or professional competencies.

Future research should adopt more fine-grained approaches to better understand how different types of technology use and occupational contexts shape technology affinity over time.

### Implications for health professions education and policy

Technology affinity may serve as a useful indicator of digital readiness within broader workforce development strategies. While infrastructure and system usability remain critical, our findings emphasize that personal digital orientation appears to play an important role in adoption. In Germany, national strategies such as the digital strategy for health and nursing care “*Digitalstrategie für das Gesundheitswesen und die Pflege”* and the Hospital Future Act *“Krankenhauszukunftsgesetz (KHZG*)” primarily emphasize infrastructure, interoperability, and clinical IT systems. However, studies suggest that workforce-related aspects such as training, user support, and participatory implementation processes have received comparatively little attention [32]. The European Health Data Space (EHDS), enacted in 2025, establishes cross-border frameworks for electronic health data exchange. This regulatory advance implies increasing expectations for health professionals’ competence in secure data handling and interoperability standards, highlighting the need for aligned educational strategies [33]. Recent updates to the Professional Qualifications Directive (2013/55/EU) mandate minimum digital knowledge and skills for nurses and other regulated health professionals across the EU, requiring Member States to implement these standards by May 2026. This reflects an emerging regulatory expectation that digital competence be formally integrated into professional qualifications and education pathways.

To translate these findings into practice, implementation strategies should be embedded at multiple levels. Educational institutions are responsible for integrating structured, experience-based digital learning opportunities into curricula, for example through longitudinal modules, simulation-based training, and the routine use of digital tools within teaching and assessment. Healthcare organizations should create supportive environments that enable regular interaction with digital systems in everyday clinical workflows, including access to user-friendly technologies, protected training time, and on-site support structures. Leadership plays a critical role in defining clear objectives for digital transformation, allocating resources, and fostering a culture that encourages experimentation and continuous skill development. Rather than relying solely on generic training programs, targeted interventions should be tailored to differences in prior exposure, confidence, and professional context.

Integrating affective readiness into implementation and educational planning may enable a shift from uniform training approaches toward differentiated capacity-building strategies that reflect professionals’ confidence levels, prior exposure, and patterns of digital engagement. This may include the use of structured assessment tools to identify varying levels of digital readiness and to guide the allocation of tailored training formats. Rather than assuming homogeneous learning needs, structured affinity profiling could inform tiered educational pathways within both undergraduate curricula and continuing professional development programs. In our sample, technology affinity varied systematically across age groups, suggesting that some professionals may benefit from advanced, application-oriented digital learning formats, whereas others may require foundational competence-building interventions that strengthen digital confidence and reduce technology-related anxiety. Such differentiation aligns with evidence highlighting the importance of organizational support structures, context-sensitive education formats, and digital leadership in fostering sustainable digital readiness among healthcare professionals [28, 29, 34]. Moreover, embedding affinity-informed strategies within Germany’s federal health governance framework may support regionally adapted digitalization initiatives that account for workforce heterogeneity while maintaining national strategic coherence.

## Data Availability

The data that support the findings of this study are available from the corresponding author upon reasonable request. The data are not publicly available due to privacy and ethical restrictions related to participant confidentiality.
